# Association of age and severe injury in young motorcycle riders: A cross-sectional study from Karachi, Pakistan

**DOI:** 10.1016/j.injury.2022.04.017

**Published:** 2022-04-20

**Authors:** Uzma Rahim Khan, Junaid A Razzak, Rashid Jooma, Martin Gerdin Wärnberg

**Affiliations:** a Department of Global Public Health, Karolinska Institutet, Stockholm, Sweden; b Department of Emergency Medicine, Aga Khan University, Karachi, Pakistan; c Department of Emergency Medicine, New York Presbyterian Weill Cornell Medicine, New York City, USA; d Centre of Excellence for Trauma and Emergencies, Aga Khan University, Karachi, Pakistan; e Department of Surgery, Aga Khan University, Pakistan; f Function Perioperative Medicine and Intensive Care, Karolinska University Hospital, Solna, Sweden

**Keywords:** Road traffic injury, Underage driving, Motorcycle riders, Pakistan, Low middle-income country

## Abstract

**Introduction:**

The burden imposed by motorcyclist deaths and injuries is high in low- and middle-income countries. Many injured motorcycle riders in these settings are underage. The aim of this study was to assess the association between age and severe injury in young motorcycle riders.

**Methods:**

We analysed road traffic injury surveillance data from the emergency rooms of five hospitals in Karachi from 2007 to 2015. We used logistic regression to assess the association of motorcycle riders’ age, categorised as underage (13–17 years), early licensing age (18–19 years) and late licensing age (20–24 years), with severe injury, defined as an Injury Severity Score (ISS) ≥ 16.

**Results:**

The study sample included 45,366 motorcycle riders. There were 10115 (22.3%) motorcycle riders aged 13–17 years, 9899 (21.8%) aged 18–19 years and 25352 (55.9%) aged 20–24 years. Almost all were male (99%). Being aged 13–17 years (adjusted odds ratio 1.25; 95% CI 1.11, 1.42) and 18–19 years (adjusted odds ratio 1.26; 95% CI 1.10, 1.43) were associated with higher odds of severe injury compared with being aged 20–24 years.

**Conclusion:**

Motorcycle riders who presented to the hospital with injuries after road traffic crashes and were aged 13–17 years and 18–19 years had significantly higher odds of severe injury than those aged 20–24 years.

## Introduction

Approximately 200,112 young people aged 10–24 years died from road traffic injuries in 2019, the most common cause of death in this age group [1]. Of the total number of road traffic deaths in this age group, 51,353 (26%) deaths were associated with motorcycle riding [1]. An overwhelming 94% of deaths of motorcyclists occurred in low- and middle-income countries, and 84% of those involved were male [1].

Adolescents are thought to be vulnerable to road traffic crashes due to limited experience and more risk-taking behaviours [2,3]. Underage, adolescent drivers are involved in fatal crashes three times more often than adults [4]. The number of road traffic crashes per million miles driven is six times higher in adolescents than in adults [5].

In most countries, the minimum driving age is 18 years, but many adolescents start to drive earlier than the legal age if they have access to household vehicles [6,7]. Underage driving is linked to adolescents’ desire for independence and adventure, augmented by peer pressure [8].

Demographic and socioeconomic factors as well as behaviours and consequences related to road crashes among adolescent car drivers have been studied in high-income countries (HICs) [9,10]. The common crash risks among adolescent car drivers in HICs are speeding, violation of safety rules, driving under the influence and use of a cell phone [11,12]. Many low-income settings lack strin- gent rules for obtaining a driver’s licence. Previous studies have re- ported high car crash rates in the period after obtaining a licence regardless of the age at licensing [2,13–18]. The risk of crashing is particularly high in the first 12 to 18 months of independent driving after obtaining a licence [19]. Graduate driver licence programmes in some HICs aim to restrict road traffic exposure in adolescent drivers and have been shown to be successful in reducing fatal crashes in young driver populations [20].

The contribution of underage drivers to the crash burden in low- and middle-income settings is unclear. Studies from many Southeast Asian countries show that boys as young as eight years have been reported to drive motorcycles [21–25]. Underage riders rarely wear helmets and are often involved in crashes [23]. Understanding underage motorcycle driving may be critical in the development of preventive measures in low-income settings, as these countries account for approximately 90% of road deaths involving adolescents globally [26].

The association of severe injuries with age of motorcycle rid- ers was inconsistent in previous studies; they found either no association with age or an association with either younger or older riders [27–31]. The aim of this study was to assess the association between age and severe injury in young motorcycle riders. The hypothesis of this study was that underage motorcycle riders aged 13–17 years and motorcycle riders of an early licensing age of 18– 19 years were more likely to have severe injuries compared to motorcycle riders aged 20–24 years.

## Methods

### Study design

It was a cross sectional study. We analysed data from a prospective road traffic injury surveillance project established in five major emergency departments in Karachi, Pakistan, from 2007–2015. We have previously reported the methodology of the Road Traffic Injury Research Project (RTIRP) [32].

### Study setting

Karachi is a largest city of Pakistan with an estimated popula- tion of 18 million people and a total road network length of over 8,000 km [33]. As previously reported, most of the injured in the city were brought to one of the three government hospitals’ emer- gency department (ED) while some also presented to large tertiary care private hospitals. The road traffic injury surveillance project was piloted in late 2006 and formally launched in 2007 and in- cluded data collection at three government and two private ter- tiary care hospitals. These hospitals receive nearly all major trauma cases in the city. Almost 98% of the study patients were enrolled from the three government hospitals.

### Study participants

For this study, we analysed data on motorcycle riders aged 13–24 years who were admitted to the ED due to road traffic injury from 2007 to 2015.

### Variables

The outcome was severe injury defined as an injury severity score (ISS) ≥ 16, as per the convention used to define major trauma [34,35].

The exposure was age with three categories; those between the ages of 13–17 years were categorised as underage, those between the age of 18–19 years as early licensing age and those 20–24 years as late licensing age [8,17].

We also used the covariates: Sex, Profession of injured riders (students versus professionals those who were earning), Time of the crash (night time versus day time), day of the crash (weekday versus weekend), month of the crash (summer versus winter), road structure (intersection versus midblock), crash location (within the city versus outside the city including highways), helmet use (helmet versus no helmet), patient transfer vehicle, hospital, injured body regions and outcome of patients in the emergency department (death vs. alive).

### Data sources/measurement

A collaborative group of health professionals, engineer and pol- icy maker developed the surveillance data collection form by us- ing WHO injury surveillance guidelines [36]. The questionnaire was piloted before it was implemented. In the three government EDs responsible for 98% of all cases, data was collected prospectively 24/7 by data collectors who worked in three eight hour shifts. In two other EDs, with better patient documentation and lower pa- tient volumes, data was collected every day from patients pre- senting with RTIs in the prior 24 h period. For majority of the patients, the research assistant collected demographic information and details of the crash by interviewing either victims, their rela- tives, ambulance drivers or other eyewitnesses. Outcome data was collected from medical record. All data was recorded on a paper forms and rechecked for consistency and completion by a data manager and entered into MS Access database on the following day except holidays when data was entered on the subsequent working days.

### Sample size

We did not estimate a sample size a priori and included all participants in the surveillance project who fit our eligibility criteria.

The sample size requirement for the multivariable logistic regression analysis has been described as between 10 and 25 events of outcomes per parameter and at least as many non-events per parameter in the multivariable logistic model [37]. The sample size of this study meets the requirements of events (severe injury) and non-events (non-severe injury).

### Data analysis

We performed the analysis using R [38]. All variables were categorical and are described using frequencies and percentages. We excluded cases for which complete information was not available. Some variables, such as vehicles involved in crashes and types of collisions (head-on, rear-end, etc.), were not included in the analysis due to 50% or more missing data (Supplemental Table 1).We used logistic regression to assess the unadjusted and adjusted associations of age group (13–17 years and 18–19 years compared with 20–24 years) and severe injury (ISS ≥ 16), estimated 95% confidence intervals (CIs), and interpreted the differences with confidence intervals that excluded no difference as statistically significant. All the covariates except injured body region and death were adjusted for in the model.

## Results

There were a total of 293,318 patients with all types of road traffic injuries in the surveillance project data. The total number of injured motorcycle riders of all ages was 132,964 (45%). Out of all motorcycle riders included in the data, 56,632 were aged 13–24 years. After removing patients with missing values, the final study sample included 45,366 injured motorcycle riders.

Table 1 shows the descriptive characteristics of the young motorcycle riders in the three age groups. There were 10,115 (22.6%) motorcycle riders aged 13–17 years, 9,899 (21.9%) aged 18–19 years and 25,352 (55.5%) aged 20–24 years. Almost all were male (99%). The majority (77%) of motorcycle riders were students in the 13–17 year age group; more than half (55%) in the 18–19 year age group were students, whereas the majority (73%) in the 20–24 year age group were professionals. Helmet use was very low in all age groups: 1.6%, 2% and 5% in the 13–17 year, 18–19 year and 20–24 year age groups, respectively. More than 70% of crashes occurred in midblock locations in all three age groups. More than 70% of riders had external injuries, more than 47% had extremity injuries, and more than 30% had head and face injuries in all three age groups. Around 472, 464 and 1063 had severe injuries, and 132, 131 and 352 died in the emergency department in 13–17 years, 18–19 and 20–24 year age groups respectively.

Table 2 shows the unadjusted and adjusted associations of age group with severe injury (ISS => 16). The odds of severe injury were higher in the 13–17 year age group (unadjusted OR 1.12; 95% CI 1.0, 1.26 and aOR 1.25 95% CIs 1.11, 1.42) and in the 18–19 year age group (unadjusted OR 1.12; 95% CIs 1.0, 1.25, and aOR 1.26; 95% CIs 1.10, 1.43) than in the 20–24 year age group. The odds of severe injury were significantly higher for those who were professionals than for students in the adjusted analyses (aOR 1.19; 95% CIs 1.07, 1.33).

The associations of sex, time of the crash, day of the crash, sea- son and road structure with severe injury were not statistically sig- nificant in the adjusted analysis. The odds of severe injuries were higher in crashes that occurred outside the city (unadjusted OR 3.3; 95% CIs 2.83, 3.9 and aOR 1.66; 95% Cis 1.38, 1.99).

In the unadjusted analysis, the odds of severe injury were significantly lower in riders who did not wear helmets than in those who wore helmets (OR 0.4; 95% CIs 0.34, 0.48). In the adjusted analysis, the odds were significantly higher in those who did not wear helmets (aOR 1.47; 95% CIs 1.18, 1.86). Pa- tient transfer vehicle type and hospital were significantly asso- ciated with severe injury in both the unadjusted and adjusted analyses.

## Discussion

This study focused on young motorcycle riders, and to the best of our knowledge, it reports the largest number of injured underage riders (13–17 years) from data collected over 9 years. Our study showed that motorcycle riders aged 13–17 years and motorcycle riders aged 18–19 years had significantly higher odds of severe injury than motorcycle riders aged 20–24 years.

The higher odds of severe injury in the 13–17-year and 18–19-year age groups might be due to limited experience in driving, as they might have been concurrently riding and learning [5]. However, riders aged 18–19 years, who are of legal age to obtain a licence, may have experienced a sense of achievement by obtaining their driving licence, which may have increased their confidence. This confidence might have led to aggressive driving, resulting in severe injury. While we did not study the driving skill and awareness of the riders related to road safety, prior studies have shown that drivers’ education is often limited to learning driving skills, without much emphasis on understanding rules and regulations [39,40].

In contrast, many of the riders in the 18–19 year age group may not have obtained a licence; thus, they may be unaware of local traffic rules and regulations, which may have resulted in road crashes. Those aged 20–24 years may have more experience driving, leading to improved judgement of road-related risks.

The evidence from previous studies was inconsistent with regards to the association of age with severe injury [28–31]. Few studies found that younger riders were associated with severe injury and mortality [28,29]. The age in these studies was catego- rized as 19 years and less, 20–24 years and 25 years and over. While the other two studies reported that older drivers were more prone to severe injuries [30,31]. These two studies used age groups 18 years or younger, 19 to 55 years, more than 55 years and 20–39 years, 40–59 years, and 60 years and older. Moreover, the compar- ison is difficult due to the variability in age groups and in the def- inition of severity. Some of the studies used injury severity scores but with different criteria for severity [29,30]. One study used self- perceived severity [28] while other used physician’s definition of injury [31].

There are various socioeconomic factors that may explain why underage motorcycle driving is common in Pakistan. Motorcycles are accessible and inexpensive modes of transport for the lower-middle class while alternative and convenient transportation op- tions are very limited in Karachi city [33]. In Pakistan, the road environment does not support safe walking for pedestrians. There are no traffic signals for pedestrian crossings. There are few walk- ways and zebra crossings for pedestrians. Moreover, motor vehicle drivers also do not give way to pedestrians in road crossings. This environment increases the threats to pedestrian safety and discour- ages walking, which in turn may lead to short, quick market trips on motorcycles, usually by underage boys. Leisure trips by thrill-seeking underage riders have also been reported; this may be due to the general lack of recreational activities in Pakistan. Many underage riders drive women members of their family on motorcycles because motorcycle driving is not considered safe or modest for women; therefore, women generally act as passengers [41].

The high number of injured underage motorcyclists indicates gaps in the enforcement of driving in licensing age. The underage motorcyclists without proper training and experience are much more vulnerable to speeding, distractions and lower helmet use. There are contextual reasons for the need of early independent mobility by young motorcyclists therefore early licensing age from existing legal age 18 years should be considered with mandatory driving education, skills and helmet use. Intervention such as graduate licensing which restricts highway driving, night time driving and unsupervised driving might be tested for young motorcyclists [42].

## Limitations

There are some limitations in this study. First, the data are over 5 years old, but the prospective data collection method from five hospitals in a lower middle-income country is valuable given that electronic data are not an option in public hospitals, where the bulk of motorcycle injuries appear. Second, although all the study participants were supposed to be enrolled in the study during the study period, there is a possibility that some of the study participants were missed by the research team due to the high volume of patients. Third, 20% of the data were missing, and patients with missing data were removed from the analysis as we attempted a complete case analysis. However, even removing missing data from such a large sample size may have a minimal effect on the power to detect true effect sizes. Fourth, age was assumed as a proxy for driving experience as well as for obtaining a driving licence in our analysis, as we did not have information about actual driving experience or the status of drivers’ licences. For example, motorcy- cle riders who were 18 years of age may have just started driv- ing or may have had several years of experience due to underage driving. We recommend that driving experience and licence status should be included to fill this gap in knowledge in future studies. Fifth, underage riders and those without licences may be underreported to hospitals due to the medicolegal requirement of physical injury cases in Pakistan. There is a possibility that this selection bias could be high for minor injuries, as severely injured patients are required to be moved to a hospital. However, in our sample, all three groups were comparable, and we obtained data both from private and public hospitals to improve the capture of patients. Sixth, there might be errors in classifying abbreviated injury score (AIS) in cases of multiple minor injuries when number of anatomic body regions involved. Injury severity score (ISS) is calculated by taking three highest AIS therefore missing minor injuries had no impact on ISS and the best efforts were made to reinforce the reg- ular training of staff. Last, we did not use mortality as an outcome as we did not record mortality that happened in other wards of hospital. We only had mortality in Emergency Department where the patient tend to stay for very brief time which might underes- timate true effect of hospital mortality.

## Conclusion

Motorcycle riders aged 13–17 years and 18–19 years had significantly higher odds of severe injury after road crashes than those aged 20–24 years. Young motorcycle riders are vulnerable to injuries, and it is important to understand their circumstances and public health and law enforcement interventions need to be implemented to prevent lifelong impacts of crashes on victims and their families.

## Supplementary Material

Supplementary table

Supplementary material associated with this article can be found, in the online version, at doi:10.1016/j.injury.2022.04.017.

## Figures and Tables

**Table 1:** Characteristics of underage motorcyclists versus young motorcycle riders of legal driving age (n=45,366).

Variables	13–17 years n = 10115 (%)	18–19 years n = 9899 (%)	20–24 years n = 25352 (%)
Sex			
Male	10064 (99.5)	9843 (99.4)	25183 (99.3)
Female	51 (0.5)	56 (0.6)	169 (0.7)
Profession			
Student	7831 (77.4)	5455 (55.1)	6973 (27.5)
Professional	2284 (22.6)	4444 (44.9)	18379 (72.5)
Time of crash			
Daytime	6041 (59.7)	5278 (53.3)	14541 (57.4)
Nighttime	4074 (40.3)	4621 (46.7)	10811 (42.6)
Day of the crash			
Weekday	6675 (66.0)	6518 (65.8)	17247 (68.0)
Weekend	3440 (34.0)	3381 (34.2)	8105 (32.0)
Season			
Winter	3148 (31.1)	3135 (31.7)	7857 (31.0)
Summer	6967 (68.9)	6764 (68.3)	17495 (69.0)
Road Structure			
Intersection	2521 (24.9)	2692 (27.2)	7321 (28.9)
Midblock	7594 (75.1)	7207 (72.8)	18031 (71.1)
Crash Location			
Inside city	9796 (96.8)	9537 (96.3)	24568 (96.9)
Outside city	319 (3.2)	362 (3.7)	784 (3.1)
Helmet use			
Yes	161 (1.6)	236 (2.4)	1194 (4.7)
No	9954 (98.4)	9663 (97.6)	24158 (95.3)
Patient transfer vehicle			
Ambulance	2285 (22.6)	2311 (23.3)	6669 (26.3)
Police	14 (0.1)	26 (0.3)	52 (0.2)
Private	7697 (76.1)	7429 (75.0)	18257 (72.0)
Public	112 (1.1)	119 (1.2)	340 (1.3)
Others	7 (0.1)	14 (0.1)	34 (0.1)
Hospital			
1	3469 (34.3)	3658 (37.0)	9391 (37.0)
2	272 (2.7)	204 (2.1)	552 (2.2)
3	13 (0.1)	9 (0.1)	30 (0.1)
4	3851 (38.1)	3160 (31.9)	8382 (33.1)
5	2510 (24.8)	2868 (29.0)	6997 (27.6)
Body region injured (multiple responses)			
Head injury	3439 (34.0)	3563 (36.0)	9040 (35.7)
Face injury	3072 (30.4)	3244 (32.8)	8003 (31.6)
Extremity injury	4819 (47.6)	4850 (49.0)	12151 (47.9)
Abdominal injury	397 (3.9)	380 (3.8)	1048 (4.1)
Chest injury	35 (0.3)	24 (0.2)	71 (0.3)
Spinal injury	241 (2.4)	240 (2.4)	662 (2.6)
External injury	7425 (73.4)	7448 (75.2)	18918 (74.6)
Injury severity score (ISS)			
Less than 16	9643 (95.3)	9435 (95.3)	24289 (95.8)
More than or equal to 16	472 (4.7)	464 (4.7)	1063 (4.2)
Outcome in emergency department			
Survival	9983 (98.7)	9768 (98.7)	25000 (98.6)
Death	132 (1.3)	131 (1.3)	352 (1.4)

**Table 2:** Unadjusted and adjusted associations of age with severe injury in young motorcycle riders (n=45,366).

Variables	Severe injury (ISS < 16) n = 43367	Severe injury (ISS > = 16) n = 1999	Unadjusted OR (95% CIs)	Adjusted OR (95% CIs)
Age group				
20–24 years	24289 (56.0)	1063 (53.2)	1	1
18–19 years	9435 (21.8)	464 (23.2)	1.12 (1.0, 1.25)	1.26 (1.10, 1.43)
13–17 years	9643 (22.2)	472 (23.6)	1.12 (1.0, 1.26)	1.25 (1.11, 1.42)
Sex				
Male	43109 (99.4)	1981 (99.1)	1	1
Female	258 (0.6)	18 (0.9)	1.52 (0.91, 2.38)	1.69 (0.97, 2.76)
Profession				
Student	19401 (44.7)	858 (42.9)	1	1
Professional	23966 (55.3)	1141 (57.1)	1.08 (0.98, 1.18)	1.19 (1.07, 1.33)
Time of crash				
Daytime	24660 (56.9)	1200 (60.0)	1	1
Nighttime	18707 (43.1)	799 (40.0)	0.88 (0.8, 0.96)	0.98 (0.89,1.08)
Day of the crash				
Weekday	29087 (67.1)	1353 (67.7)	1	1
Weekend	14280 (32.9)	646 (32.3)	0.97 (0.88, 1.07)	1.01 (0.91, 1.12)
Season				
Winter months	13510 (31.2)	630 (31.5)	1	1
Summer months	29857 (68.8)	1369 (68.5)	0.98 (0.89, 1.08)	0.99 (0.90, 1.10)
Road structure				
Intersection	12102 (27.9)	432 (21.6)	1	1
Midblock	31265 (72.1)	1567 (78.4)	1.4 (1.26, 1.57)	1.10 (0.99, 1.24)
Crash location				
Inside city	42086 (97.0)	1815 (90.8)	1	1
Outside city	1281 (3.0)	184 (9.2)	3.3 (2.83, 3.9)	1.66 (1.38, 1.99)
Helmet use				
Yes	1435 (3.3)	156 (7.8)	1	1
No	41932 (96.7)	1843 (92.2)	0.4 (0.34, 0.48)	1.47 (1.18, 1.86)
Patient transfer vehicle				
Ambulance	10086 (23.3)	1179 (59.0)	1	1
Police	82 (0.2)	10 (0.5)	1.04 (0.51, 1.92)	1.26 (0.59, 2.40)
Private	32629 (75.2)	754 (37.7)	0.2 (0.18, 0.22)	0.27 (0.25, 0.30)
Public	518 (1.2)	53 (2.7)	0.88 (0.65, 1.16)	1.33 (0.98, 1.77)
Others	52 (0.1)	3 (0.2)	0.49(0.12, 1.34)	0.70 (0.17, 1.91)
Hospital				
1	15745 (36.3)	773 (38.7)	1	1
2	608 (1.4)	420 (21.0)	14.07(12.18, 16.24)	11.71 (9.84, 13.95)
3	44 (0.1)	8 (0.4)	3.7 (1.61, 7.47)	3.02 (1.28, 6.30)
4	14978 (34.5)	415 (20.8)	0.56 (0.5, 0.64)	0.67 (0.59, 0.75)
5	11992 (27.7)	383 (19.2)	0.65 (0.57, 0.74)	0.83 (0.73, 0.94)

## References

[1] IHME. Global Burden of Disease Collaborative Network. Global Burden of Disease Study 2019 (GBD 2019). Seattle, United States of America: Institute for Health Metrics and Evaluation (IHME); 2021.

[2] Gershon P, Ehsani JP, Zhu C, Sita KR, Klauer S, Dingus T, Crash risk and risky driving behavior among adolescents during learner and independent driving periods. J. Adolesc. Health 2018;63:568–74. 30006026 10.1016/j.jadohealth.2018.04.012

[3] Sarkar S, Andreas M. Acceptance of and engagement in risky driving behaviors by teenagers. Adolescence 2004;39:687. 15727407

[4] Walshe EA, Ward McIntosh C, Romer D, Winston FK. Executive function capacities, negative driving behavior and crashes in young drivers. Int. J. Environ. Res. Public Health 2017;14:1314. 29143762 10.3390/ijerph14111314 PMC5707953

[5] Banz BC, Fell JC, Vaca FE. Focus: death: complexities of young driver injury and fatal motor vehicle crashes. Yale J. Biol. Med 2019;92:725. 31866787 PMC6913817

[6] Shults RA, Banerjee T, Perry T. Who’s not driving among US high school se- niors: A closer look at race/ethnicity, socioeconomic factors, and driving status. Traffic Inj. Prev 2016;17:803–9. 27064697 10.1080/15389588.2016.1161761 PMC5712435

[7] Zamani-Alavijeh F, Niknami S, Bazargan M, Mohamadi E, Montazeri A, Ghofranipour F, Risk-taking behaviors among motorcyclists in middle east countries: a case of islamic republic of Iran. Traffic Inj. Prev 2010;11:25–34. 20146140 10.1080/15389580903330355

[8] Alderman EM, Johnston BD. The teen driver. Pediatrics 2018:142. 10.1542/peds.2018-2163 30249622

[9] Bates LJ, Davey J, Watson B, King MJ, Armstrong K. Factors contributing to crashes among young drivers. Sultan Qaboos Univ. Med. J 2014;14:e297. 25097763 PMC4117653

[10] Hanna CL, Hasselberg M, Laflamme L, Möller J. Road traffic crash circumstances and consequences among young unlicensed drivers: a Swedish cohort study on socioeconomic disparities. BMC Public Health 2010;10:1–8. 20074325 10.1186/1471-2458-10-14 PMC2820473

[11] Boulagouas W, García-Herrero S, Chaib R, Febres JD, Mariscal MÁ, Djebabra M. An investigation into unsafe behaviors and traffic accidents involving unli- censed drivers: a perspective for alignment measurement. Int. J. Environ. Res. Public Health 2020;17:6743. 32947935 10.3390/ijerph17186743 PMC7560083

[12] Jewett A, Shults RA, Bhat G. Parental perceptions of teen driving: restrictions, worry and influence. J. Saf. Res 2016;59:119–23. 10.1016/j.jsr.2016.09.003 PMC5201134 27846995

[13] Ehsani JP, Bingham CR, Shope JT. The effect of the learner license graduated driver licensing components on teen drivers’ crashes. Accid. Anal. Prev 2013;59:327–36. 23856640 10.1016/j.aap.2013.06.001

[14] Lewis-Evans B Crash involvement during the different phases of the New Zealand graduated driver licensing system (GDLS). J. Saf. Res 2010;41:359–65. 10.1016/j.jsr.2010.03.006 20846552

[15] Masten SV, Foss RD, Marshall SW. Graduated driver licensing and fatal crashes involving 16-to 19-year-old drivers. JAMA 2011;306:1098–9. 21917580 10.1001/jama.2011.1277

[16] Mayhew DR, Simpson HM, Pak A. Changes in collision rates among novice drivers during the first months of driving. Accid. Anal. Prev 2003;35:683–91. 12850069 10.1016/s0001-4575(02)00047-7

[17] McCartt AT, Mayhew DR, Braitman KA, Ferguson SA, Simpson HM. Effects of age and experience on young driver crashes: review of recent literature. Traffic Inj. Prev 2009;10:209–19. 19452361 10.1080/15389580802677807

[18] Simons-Morton BG, Ouimet MC, Zhang Z, Klauer SE, Lee SE, Wang J, Crash and risky driving involvement among novice adolescent drivers and their parents. Am. J. Public Health 2011;101:2362–7. 22021319 10.2105/AJPH.2011.300248 PMC3222425

[19] Curry AE, Metzger KB, Williams AF, Tefft BC. Comparison of older and younger novice driver crash rates: Informing the need for extended Graduated Driver Licensing restrictions. Accid. Anal. Prev 2017;108:66–73. 28858774 10.1016/j.aap.2017.08.015

[20] Tefft BC, Williams AF, Grabowski JG. Driver licensing and reasons for delaying licensure among young adults ages 18–20 United States 2012. Injury Epidemiol. 2014;1:1–8. 10.1186/2197-1714-1-4 PMC4981029 27747671

[21] Lutfi AZ. The phenomenon of underage motorbike riders in junior high school students: a critical review of juvenile delinquency. J. Indones. Soc. Sci. Hu- manit 2020;10:121–34.

[22] Pervez A, Lee J, Huang H. Identifying factors contributing to the motorcycle crash severity in Pakistan. J. Adv. Transp 2021;2021:6636130.

[23] Piyapromdee U, Adulyanukosol V, Lewsirirat S. Increasing road traffic injuries in underage motorcyclists. Thai J. Orthop. Surg 2015;39:3–7.

[24] Rahman NH, Rainis R, Noor SH, Mohamad SMS. The Buffering analysis to identify common geographical factors within the vicinity of severe injury related to motor vehicle crash in Malaysia. World J. Emerg. Med 2016;7:278. 27942345 10.5847/wjem.j.1920-8642.2016.04.007 PMC5143312

[25] Rathinam C, Nair N, Gupta A, Joshi S, Bansal S. Self-reported motorcycle riding behaviour among school children in India. Accid. Anal. Prev 2007;39: 334–339. 17049470 10.1016/j.aap.2006.09.002

[26] Nantulya VM, Reich MR. Equity dimensions of road traffic injuries in low-and middle-income countries. Inj. Control Saf. Promot 2003;10:13–20. 12772481 10.1076/icsp.10.1.13.14116

[27] Zambon F, Hasselberg M. Factors affecting the severity of injuries among young motorcyclists a Swedish nationwide cohort study. Traffic Inj. Prev 2006;7:143–9. 16854708 10.1080/15389580600555759

[28] Rutter DR, Quine L. Age and experience in motorcycling safety. Accid. Anal. Prev 1996;28:15–21. 8924181 10.1016/0001-4575(95)00037-2

[29] Mullin B, Jackson R, Langley J, Norton R. Increasing age and experience: are both protective against motorcycle injury? A case-control study. Inj. Prev 2000;6:32–5. 10728539 10.1136/ip.6.1.32 PMC1730576

[30] Talving P, Teixeira PG, Barmparas G, Dubose J, Preston C, Inaba K, Motor- cycle-related injuries: effect of age on type and severity of injuries and mor- tality. J Trauma 2010;68:441–6. 20154556 10.1097/TA.0b013e3181cbf303

[31] Jackson TL, Mello MJ. Injury patterns and severity among motorcyclists treated in US emergency departments, 2001–2008: a comparison of younger and older riders. Inj. Prev 2013;19:297–302. 23393164 10.1136/injuryprev-2012-040619

[32] Razzak JA, Shamim MS, Mehmood A, Hussain SA, Ali MS, Jooma R. A successful model of road traffic injury surveillance in a developing country: process and lessons learnt. BMC Public Health 2012;12:1–5. 22591600 10.1186/1471-2458-12-357 PMC3508990

[33] Hoor-Ul-Ain S An empirical review of Karachi’s transportation predicaments: a paradox of public policy ranging from personal attitudes to public opinion in the megacity. J. Transp. Health 2019;12:164–82.

[34] Schröter C, Urbanek F, Frömke C, Winkelmann M, Mommsen P, Krettek C, Injury severity in polytrauma patients is underestimated using the injury severity score: a single-center correlation study in air rescue. Eur. J. Trauma Emerg. Surg 2019;45:83–9. 29234837 10.1007/s00068-017-0888-1

[35] VanDerHeyden N, Cox TB. Trauma scoring. In: Current Therapy of Trauma and Surgical Critical Care. Elsevier; 2008. p. 26–32.

[36] World Health Organization. Injury surveillance guidelines: World Health Organization; 2001.

[37] Pajouheshnia R, Pestman WR, Teerenstra S, Groenwold RH. A computational approach to compare regression modelling strategies in prediction research. BMC Med. Res. Method 2016;16:1–10. 10.1186/s12874-016-0209-0 PMC4997720 27557642

[38] Team R Core. R: a language and environment for statistical computing [Internet]. Vienna, Austria: R Foundation for Statistical Computing; 2020. 2017.

[39] Batool Z, Carsten O, Jopson A. Road safety issues in Pakistan: a case study of Lahore. Transp. Plann. Technol 2012;35:31–48.

[40] Hussain M, Shi J. Effects of proper driving training and driving license on aberrant driving behaviors of Pakistani drivers-a proportional odds approach. J. Transp. Saf. Secur 2019;13:1–19.

[41] Adeel M, Anthony GO Y, Zhang F. Gender, mobility and travel behavior in Pakistan: Analysis of 2007 Time Use Survey. 2013. https://mpra.ub.uni-muenchen.de/55474/.

[42] Bates LJ, Allen S, Armstrong K, Watson B, King MJ, Davey J. Graduated driver licensing: an international review. Sultan Qaboos Univ. Med. J 2014;14:e432. 25364543 PMC4205052

