# Supplementary material for: Association of age and severe injury in young motorcycle riders: A cross-sectional study from Karachi, Pakistan
Source: Injury. Author manuscript; Available in PMC 2025 Jul 6. (PMC12229256; doi:10.1016/j.injury.2022.04.017)
Supplement: Supplementary table [file NIHMS1823663-supplement-Supplementary_table.pdf]

Association of age and severe injury in young motorcycle riders: A cross-sectional study from Karachi, Pakistan

Supplemental Material

Table 1
Missing data for variables of interest n =56,632

Variables	N (%)
Helmet use	2721 (4.8)
Road structure	2344 (4.14)
Profession	2318 (4.09)
Injury severity score	2159 (3.81)
Day of crash	1493 (2.64)
Time of crash	1406 (2.48)
Patient transfer vehicle	1042 (1.84)
Crash location	263 (0.46)
Outcome in emergency department	139 (0.25)
Sex	15 (0.03)
Season	0
Hospital	0
